# Targeting 14-3-3ζ Overcomes Resistance to Epidermal Growth Factor Receptor-Tyrosine Kinase Inhibitors in Lung Adenocarcinoma via BMP2/Smad/ID1 Signaling

**DOI:** 10.3389/fonc.2020.542007

**Published:** 2020-10-05

**Authors:** Jinfang Cui, Yang Song, Xuejiao Han, Jing Hu, Yanbo Chen, Xuesong Chen, Xiaomin Xu, Ying Xing, Hailing Lu, Li Cai

**Affiliations:** ^1^The Fourth Department of Medical Oncology, Harbin Medical University Cancer Hospital, Harbin, China; ^2^Department of Orthopedic Surgery, The Second Affiliated Hospital of Harbin Medical University, Harbin, China

**Keywords:** EGFR-mutant, BMP2/Smad/ID-1 signaling pathway, EGFR-TKI resistance, lung adenocarcinoma, 14-3-3ζ

## Abstract

**Background:** The 14-3-3ζ protein, which acts as a putative oncoprotein, has been found to promote the proliferation, metastasis, and chemoresistance of cancer cells in several cancers including lung adenocarcinoma (LUAD); however, its significance in epidermal growth factor receptor–tyrosine kinase inhibitor (EGFR-TKI) resistance remains unknown.

**Methods:** The Cancer Genome Atlas (TCGA) database was used to determine 14-3-3ζ expression in pancancer and LUAD. 14-3-3ζ and ID1 expression was then examined in clinical LUAD samples by immunohistochemistry (IHC). Lentiviral transfection with 14-3-3ζ-specific small hairpin RNA (shRNA) was used to establish stable 14-3-3ζ knockdown gefitinib-resistant PC9 (PC9/GR) and H1975 cell lines. The effect of 14-3-3ζ knockdown on reversing EGFR-TKI resistance was determined *in vitro* by Cell Counting Kit-8 (CCK-8), wound healing, Transwell assays, and flow cytometry. A xenograft tumor model was established to evaluate the role of 14-3-3ζ in EGFR-TKI resistance. Microarray analysis results showed multiple pathways regulated by 14-3-3ζ-shRNA.

**Results:** In the present study, we demonstrated that based on the TCGA, pancancer and LUAD 14-3-3ζ expression was elevated and predicted unfavorable prognosis. In addition, high 14-3-3ζ expression was associated with advanced T stage, TNM stage, presence of lymph node metastasis and, importantly, poor treatment response to EGFR-TKIs in LUAD patients with EGFR-activating mutations. 14-3-3ζ shRNA sensitized EGFR-TKI-resistant human LUAD cells to gefitinib and reversed epithelial-to-mesenchymal transition (EMT). After 14-3-3ζ depletion, bone morphogenetic protein (BMP) signaling activation was decreased in EGFR-TKI-resistant cells in microarray analysis, which was further validated by Western blot analysis. Furthermore, the expression of 14-3-3ζ positively correlates with ID1 expression in human EGFR-mutant LUAD patient samples. *In vivo*, there was a reduction in the tumor burden in mice treated with 14-3-3ζ shRNA and gefitinib compared to mice treated with gefitinib alone.

**Conclusion:** Our work uncovers a hitherto unappreciated role of 14-3-3ζ in EGFR-TKI resistance. This study might provide a potential therapeutic approach for treating LUAD patients harboring EGFR mutations.

## Introduction

Lung cancer remains the leading cause of cancer death worldwide, and pathologically, most lung cancer is non-small cell lung cancer (NSCLC) (1, 2). NSCLC is a heterogeneous disease typically classified into three broad subtypes: lung adenocarcinoma (LUAD), lung squamous cell carcinoma (LUSC), and large cell carcinoma (3, 4). LUAD is a devastating disease because of dismal patient survival (5). Encouragingly, epidermal growth factor receptor-tyrosine kinase inhibitors (EGFR-TKIs), such as gefitinib and erlotinib, lead to unprecedented clinical benefits and provide a new weapon against LUAD with EGFR-activating mutations (6). However, most LUAD patients eventually develop acquired resistance to EGFR-TKIs (6). The identified acquired resistance mechanisms have been mainly categorized as secondary mutations in the EGFR gene (T790M and other rare mutations), the activation of alternative signaling pathways, and phenotypic changes such as epithelial–mesenchymal transition (EMT) (7, 8). The unpredictability and diversity of EGFR-TKI resistance mechanisms present a challenge for developing innovative treatment strategies that can overcome EGFR-TKI resistance in patients (9).

The human 14-3-3 proteins share a highly conserved homology and are composed of seven isoforms (β, σ, ε, τ/θ, γ, η, and ζ) with unique expression patterns in different cell types and tissues (10). Of the seven isoforms in eukaryotic organisms, 14-3-3ζ [also known as tyrosine 3-monooxygenase/tryptophan 5-monooxygenase activation protein zeta (YWHAZ)] has been proposed to be directly involved in cellular transformation and proliferation (11). Importantly, accumulating evidence has revealed that 14-3-3ζ plays key roles in the regulation of diverse diseases, such as obesity (12), diabetes (13), and cancer (14). 14-3-3ζ functions as a central node in regulating critical processes in cancer, including cell motility, metabolism, cycle progression, mitogenicity, apoptosis, and EMT (11, 14–20). Previous studies have reported that the occurrence of EMT confers acquired resistance to EGFR-targeted therapy (21) and that 14-3-3ζ promotes the EMT phenotype in lung cancer (16). However, to the best of our knowledge, the role of 14-3-3ζ in EGFR-TKI resistance remains unknown.

Bone morphogenetic proteins (BMPs) are one of the transforming growth factor-beta (TGF-β) subfamilies, and they use similar signal transduction pathways that involve transmembrane serine threonine kinase receptors and Smad proteins (22). Among BMPs, overexpression of BMP2 was indicated to occur in ~98% of lung carcinomas and to contribute to lung cancer progression (23–25). BMP2 can trigger the phosphorylation of Smad1/5 by the binding of Smad1/5 to the BMP2 receptors, and activated Smad1/5 binds Smad4, translocates to the nucleus, binds to Smad-binding elements in the promotor of inhibitors of differentiation 1 (ID1), and induces ID1 transcription (26). ID1 promotes tumorigenesis, EMT, and metastasis of various cancer types, including lung cancer (27–30). The mechanism of regulation of the BMP2/Smad/ID1 signaling pathway still needs to be further explicated.

In the present study, we provided evidence that 14-3-3ζ inhibition significantly attenuated LUAD cells refractory to EGFR-TKIs *in vitro* and *in vivo*, and this was accompanied by EMT reversal. We performed global gene expression microarray profiling in EGFR-TKI-resistant cells after small hairpin RNA (shRNA) knockdown of 14-3-3ζ and analyzed the gene expression data. Here, we demonstrated that 14-3-3ζ has a positive regulatory effect on the BMP2/Smad/ID1 signaling pathway. Our findings suggest that 14-3-3ζ might be a novel and potential target for overcoming EGFR-TKI resistance in LUAD with an EGFR-activating mutation.

## Materials and Methods

### Study Design

The experimental design and construction scheme of this study are systematically presented in Supplementary Figure 1.

#### Bioinformatics Analysis

We used web-based tools available through GEPIA (Gene Expression Profiling Interactive Analysis, http://gepia.cancer-pku.cn/), which is based on The Cancer Genome Atlas (TCGA) and Genotype-Tissues Expression (GTEx) databases to detect 14-3-3ζ mRNA levels in various tumors (31). The online Kaplan–Meier plotter (http://kmplot.com/analysis/) tool was used to test the predictive significance of 14-3-3ζ expression (32).

### Cell Lines, Reagents, and Tissue Specimens

Human NSCLC cell lines H1975 (EGFR L858R/T790M mutations; gefitinib resistant), PC9 (EGFR with deletion of E746_A750; gefitinib sensitive), and PC9/GR (gefitinib-resistant PC9 cell line) were maintained in our laboratory, as previously reported (9, 33).

NCI-H1975 cells were cultured in RPMI 1640 media with 10% fetal bovine serum (FBS; HyClone, USA) and 1% penicillin/streptomycin (Gibco, USA). PC-9 and PC-9/GR cells were cultured in Dulbecco's modified Eagle's medium (DMEM) with 10% FBS and 1% penicillin/streptomycin. The cells were maintained at 37°C in a humidified incubator with 5% CO_2_. Cell lines were periodically authenticated by short-tandem repeat (STR) profiling using previously reported methods (34).

Clinical samples were obtained from 128 patients with LUAD who were surgically treated at Harbin Medical University Cancer Hospital from November 2009 to December 2017. These 128 LUAD patients harbored gefitinib-sensitive EGFR mutations such as exon 19 deletion and L858R or gefitinib-resistant EGFR mutations such as T790M. Of these patients, 41, who were harboring gefitinib-sensitive EGFR mutations, such as exon 19 deletion and L858R, were treated with EGFR-TKIs when LUAD progressed to stage IV. The clinicopathological characteristics of these 41 patients when they underwent surgery are shown in Supplementary Table 1.

The EGFR-TKI-insensitive group included patients with disease progression or stable disease without an extended (6 months) progression-free survival (PFS), and the EGFR-TKI-sensitive group included patients with a complete or partial response or stable disease with prolonged PFS (6 months) (35). All specimens for immunohistochemistry (IHC) were formalin fixed, paraffin embedded, and histologically examined for the presence of non-necrotic tumor areas.

Fresh tissues (paired LUAD tumor samples and matched adjacent normal tissue samples) were resected from 10 LUAD patients harboring gefitinib-sensitive EGFR mutations between August 2017 and December 2017. Normal lung tissue samples were taken from areas at a standard distance (3 cm) from resected tissues of LUAD patients who underwent surgery (36). The study protocol was carefully explained to the participants, and written informed consent was obtained from all participants. Ethical clearance and approval (No. KY2017-09) was obtained from the Ethics Committee of Harbin Medical University Cancer Hospital.

### IHC

IHC experimental procedures were performed as previously described (37). Antibodies used in IHC include anti-14-3-3ζ (Santa Cruz Biotechnology, SC-293415, diluted at 1:100) and anti-ID1 (Santa Cruz Biotechnology, SC-374287, diluted at 1:100). The results were confirmed by at least two pathologists in a double-blind manner.

### Western Blot Analysis

Western blot analysis was performed according to a previously described standard method (37). The primary antibodies used for the Western blot analyses were as follows: 14-3-3ζ (Santa Cruz Biotechnology, SC-293415, diluted at 1:500), E-cadherin (Abcam, ab133597, diluted at 1:5,000), N-cadherin (Abcam, ab76057, diluted at 1:1,000), vimentin (Abcam, ab137321, diluted at 1:1,500), BMP2 (Abcam, ab214821, diluted at 1:1,000), BMPR2 (Abcam, ab124463, diluted at 1:500), Smad1 (Cell Signaling Technology, #6944, diluted at 1:1,000), Smad5 (Cell Signaling Technology, #9517, diluted at 1:1,000), phosphorylated (p)-Smad1/5 (Cell Signaling Technology, #9516, diluted at 1:1,000), ID1 (Santa Cruz Biotechnology, SC-374287, diluted at 1:500) and β-actin (used as the loading control; Sigma, A1978). Western blot bands were quantified by ImageJ software (U.S. National Institutes of Health, USA). The experiments were repeated three times.

### Wound Healing Assay

Cells at a density of 1 × 10^6^ cells/well were seeded in six-well plates. When the cells were grown to 80 to 90% confluence, a cross-shaped wound was scratched by dragging a 10-μl sterile pipette tip across the monolayers of cells, and the cell debris were rinsed with phosphate-buffered saline (PBS) (9). The process of wound healing was then observed at 0, 24, and 48 h, and the cells were stained with crystal violet at 48 h for a clearer view of the wound. Three replicate wells were used for each condition, and three cell images per well were captured by an inverted fluorescence microscope for quantification analysis.

### Cell Migration and Invasion Assay

Cell migration and invasion assays were performed as previously described (38). Briefly, Matrigel (BD Biosciences, USA) was thawed on a plate on ice overnight in a cold room, and RPMI 1640 or DMEM was added at a ratio of 1:7. Thirty microliters of diluted Matrigel was pipetted into the upper chamber of Transwell Cell inserts (Corning, USA). The cell suspension containing 2 × 10^4^ cells/ml in serum-free RPMI 1640 medium or DMEM was prepared, of which 200 μl of the cell suspension was transferred to the upper chamber. The lower chamber contained 600 μl of complete growth medium with 10% FBS. After incubation at 37°C for 24 or 48 h in a Transwell without pre-applied Matrigel and precoated Matrigel, respectively, the inserts were fixed and stained for 15 min in 25% methanol containing 0.5% crystal violet. The number of invaded cells per field view was counted using the cell counter plugin in ImageJ. All experiments were performed in triplicate.

### Retroviral Infection and Transfection

Lentiviruses containing shRNA targeting 14-3-3ζ were purchased from Shanghai GeneChem (GeneChem Co., Ltd, Shanghai, China). The shRNA 14-3-3ζ target sequence was 5′-TCGAGAATACAGAGAGAAA-3′. H1975 and PC9/GR cells were infected with lentiviral particles and cultured in complete RPMI 1640 media or DMEM containing puromycin (Santa Cruz Biotechnology, Santa Cruz, CA) to select the 14-3-3ζ-silenced cell clones. Cells transfected with scrambled shRNA were used as controls.

### Microarray Processing and Analysis

Microarray processing and analysis were performed as previously described (9). To define the gene expression profiles, Affymetrix Human GeneChip PrimeView (Thermo Fisher Science, Catalog number: 902487) was used for microarray analysis according to the manufacturer's instructions. An array of raw data was generated by scanning with a GeneChip Scanner 3000. According to the following criteria, differentially expressed genes between NCI-H1975 cell lines transfected with 14-3-3ζ-shRNA and NCI-H1975 cell lines transfected with scrambled shRNA were selected: *p* < 0.001 and absolute fold change >4. Pathway enrichment analysis was conducted for differentially expressed genes using the commercially available Ingenuity Pathway Analysis (IPA, QIAGEN Bioinformatics) software.

### Apoptosis Assays

NCI-H1975 and PC9/GR cells were digested, washed with cold PBS, and resuspended in binding buffer, according to the instructions of the apoptosis kit. PE Annexin V and 7-amino-actinomycin (7-AAD; BD Pharmingen, San Diego, CA, USA) were added to the fixed cells for 15 min in the dark at room temperature. Then, Annexin V binding buffer was added to the mixture before the fluorescence was measured with an LSR II flow cytometer (BD, USA). Cell apoptosis was analyzed using FlowJo software (FlowJo, LLC, Bethesda, USA). Three separate experiments were performed.

### Cell Viability Assay

The viability of cells was measured using the Cell Counting Kit-8 (CCK-8; Dojindo Molecular Technologies, Kumamoto, Japan). As previously described (33), cells were seeded in 96-well flat-bottomed plates with each well-containing 7 × 10^3^ cells in 200 μl of culture medium. After 48 h of growth with different concentrations of gefitinib (0–40 μM), the cells were incubated in 10% CCK-8 solution for an additional 1 h at 37°C in the dark. The absorbance at 450 nm (A450) was examined on a microplate reader (BioTek, Winooski, VT, USA). Three parallel experiments were performed in five replicate wells per sample. The half maximal inhibitory concentration (IC_50_) values were determined using IBM SPSS Statistics 20.0 software.

### Xenograft Models

Animal experiments were performed in accordance with the Institutional Ethics Committee for the Administration of Laboratory Animals of Harbin Medical University, China. The experimental process was previously described (33). A total of 5 × 10^6^ H1975/scrambled shRNA cells (ctrl) or H1975/14-3-3ζ-shRNA cells that had been resuspended in 100 μl PBS were injected subcutaneously into the right flanks of male BALB/c nude mice (6 weeks old, 17 ± 3 g weight, Changzhou Cavens Laboratory Animal Co., Ltd.) in each group (*n* = 10 per group). Tumor volume (V) was calculated with the formula: π/6 × larger diameter × (smaller diameter)^2^. When established tumors of an approximate diameter of 75 mm^3^ were detected, the mice bearing H1975 cells with or without the stable knockdown of 14-3-3ζ were randomized into two subgroups (*n* = 4 per subgroup). The mice in each subgroup received gefitinib (50 mg/kg HY-50895, MedChemExpress Co., Ltd.) or PBS by oral gavage every day. Subsequently, xenografted tumor size was monitored every 3 days for 28 days. Tumor weight was measured after excision at 28 days of the experiment.

### Statistical Analysis

Values are expressed as the mean ± standard deviation (SD) for at least three independent experiments if the data were quantitative. The software programs SPSS 20.0 (SPSS, Chicago, IL, USA) and GraphPad Prism 5 were used to analyze the data. Continuous variables between the two groups were analyzed by Student's *t*-tests. The differences in categorical variables were analyzed with χ^2^-tests. A statistically significant difference was defined as ^*^p < 0.05, ^**^p < 0.01, or ^***^p < 0.001.

## Results

### 14-3-3ζ Expression Predicts LUAD and Pancancer Prognosis

**Figure 1:**
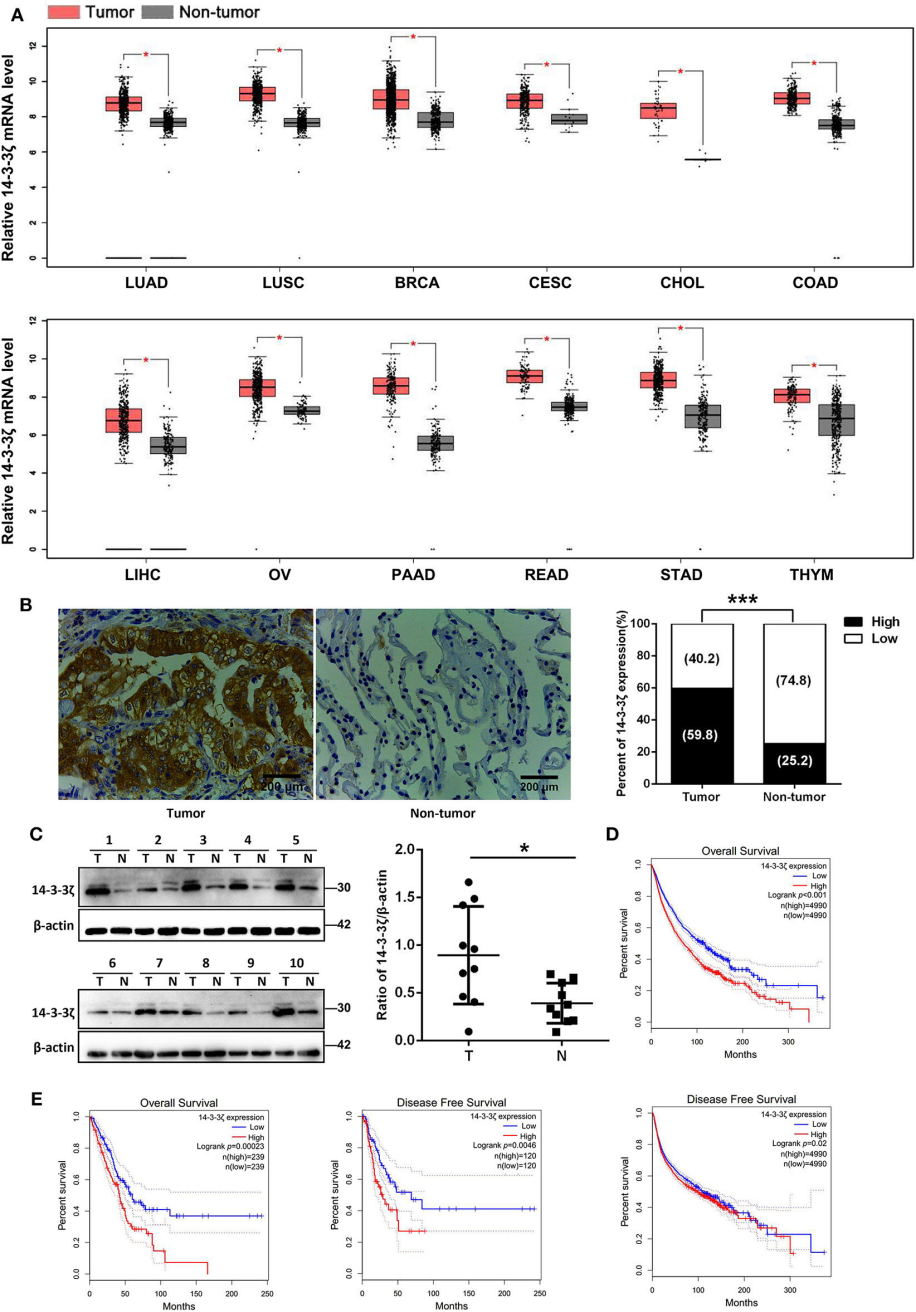
Elevated 14-3-3ζ expression positively correlates with poor prognosis of lung adenocarcinoma (LUAD) patients. **(A)** Relative mRNA levels of 14-3-3ζ in different tumors were analyzed by Gene Expression Profiling Interactive Analysis (GEPIA). *p < 0.05 **(B)** Representative immunostaining images for 14-3-3ζ expression in 128 human LUAD tissues with epidermal growth factor receptor (EGFR) mutations (tumor) and 31 non-tumoral lung tissues (non-tumor). Original magnification, ×400; Scale bar = 200 μm. Histogram showing percentages of 14-3-3ζ expression in tumor and non-tumor tissues. ***p < 0.001 **(C)** Western blot analysis of 14-3-3ζ expression in lysates originating from 10 human LUAD samples with EGFR mutant (T) and matched adjacent normal tissues (N). Right panel: quantification of relative expression levels of 14-3-3ζ. β-actin was used as a loading control. *p < 0.05. **(D)** Kaplan–Meier analysis of overall and disease-free survival (DFS) for high and low 14-3-3ζ expression levels in pancancer determined by GEPIA. **(E)** Kaplan–Meier analysis of overall and DFS for high and low 14-3-3ζ expression levels in LUAD determined by GEPIA.

We examined 14-3-3ζ expression at the mRNA level in different carcinomas based on the TCGA and GTEx databases (39). We found that 14-3-3ζ was elevated in 12 kinds of cancer tissues, including LUAD, LUSC, breast invasive carcinoma, cervical squamous cell carcinoma, cholangiocarcinoma, colon adenocarcinoma, liver hepatocellular carcinoma, ovarian serous cystadenocarcinoma, pancreatic adenocarcinoma, rectum adenocarcinoma, stomach adenocarcinoma, and thymoma tissues, compared to those in non-tumor tissues (Figure 1A). We further verified the 14-3-3ζ expression in LUAD tissues with EGFR-activating mutations using IHC. Consistently, 14-3-3ζ had a higher expression in LUAD tissues than in normal adjacent tissues (Figure 1B). By Western blot analysis, we also discovered that the 14-3-3ζ expression levels in LUAD tissues were higher than those observed in the paired non-tumoral lung tissues (Figure 1C).

The TCGA database was used to detect the prognostic significance of pancancer and LUAD 14-3-3ζ expression. From 9,980 tumors across 26 kinds of cancers, we showed that high 14-3-3ζ expression was a prognostic factor for overall survival (OS) and disease-free survival (DFS) in pancancer patients (Figure 1D). Moreover, we found that a high 14-3-3ζ expression was associated with poor survival for LUAD patients (Figure 1E). Using the online Kaplan–Meier plotter tool (40), the curves consistently depicted that patients with high 14-3-3ζ expression levels had shorter OS and postprogression survival (PPS) than those with low 14-3-3ζ expression levels (Supplementary Figure 2).

### The Clinicopathological Signature of 14-3-3ζ in LUAD With EGFR-Activating Mutations

**Figure 2:**
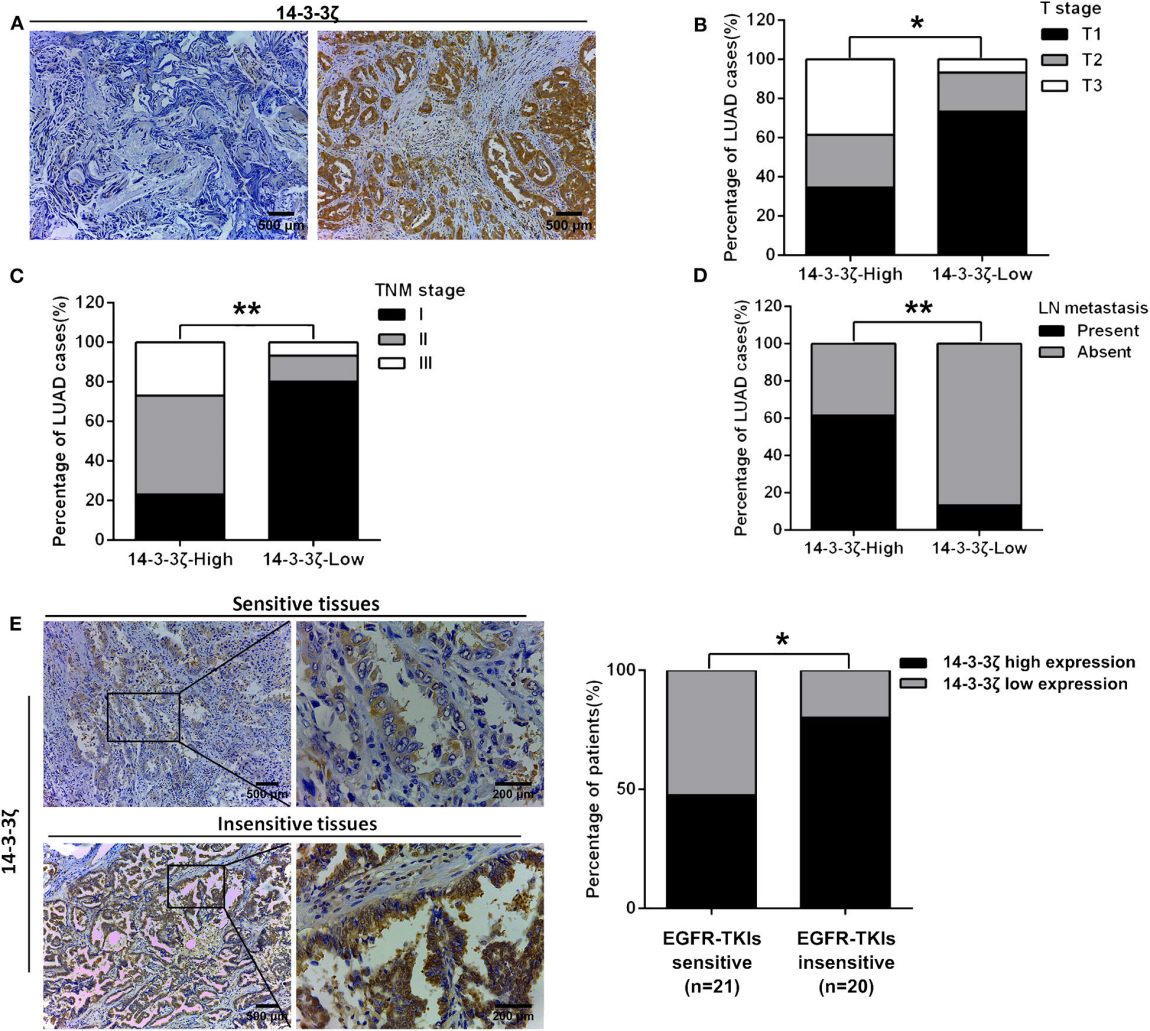
The clinicopathological signature of 14-3-3ζ in LUAD tissues with EGFR-activating mutations. **(A)** Representative immunohistochemistry (IHC) staining images of low expression (left) and high expression (right) of 14-3-3ζ in LUAD tissues. Magnification, ×100; Scale bar = 500 μm. **(B–D)** The protein expression level of 14-3-3ζ was classified as low or high based on the intensity and proportion of positively stained cells in these specimens. The percentages of patients with different T stages **(B)**, different TNM stages **(C)**, with and without lymph node metastasis, and **(D)** were assigned according to the expression level of 14-3-3ζ. **(E)** Representative immunostaining profiles of 14-3-3ζ in drug-sensitive (PFS ≥ 6 months, *n* = 21) and drug-insensitive (PFS < 6 months, *n* = 20) LUAD tissues. Magnification, ×100; Scale bar = 500 μm. Magnification, ×400; Scale bar = 200 μm (left). The percentages of patients with high expression (black bar) and low expression of 14-3-3ζ (gray bar) were assigned according to different responses to EGFR-tyrosine kinase inhibitors (TKIs) (right). *p < 0.05, **p < 0.01.

Next, we investigated the clinicopathological signature of 14-3-3ζ and the relationship between 14-3-3ζ expression and EGFR-TKI responsiveness in LUAD with EGFR-activating mutations. The protein expression level of 14-3-3ζ was classified as low or high based on the intensity and proportion of positively stained cells in these specimens (Figure 2A). Our IHC analysis results revealed that high 14-3-3ζ expression was associated with advanced T stage, advanced TNM stage, and the presence of lymph node metastasis (Figures 2B–D; Supplementary Table 2). Notably, 14-3-3ζ had a higher expression in the EGFR-TKI-refractory specimens (PFS < 6 months) than in the EGFR-TKI-sensitive specimens (PFS ≥ 6 months) by IHC (Figure 2E; Supplementary Table 1). Our results indicated that 14-3-3ζ expression could play a role in EGFR-TKI resistance.

### Knockdown of 14-3-3ζ Sensitizes Gefitinib-Resistant LUAD Cells to Gefitinib

**Figure 3:**
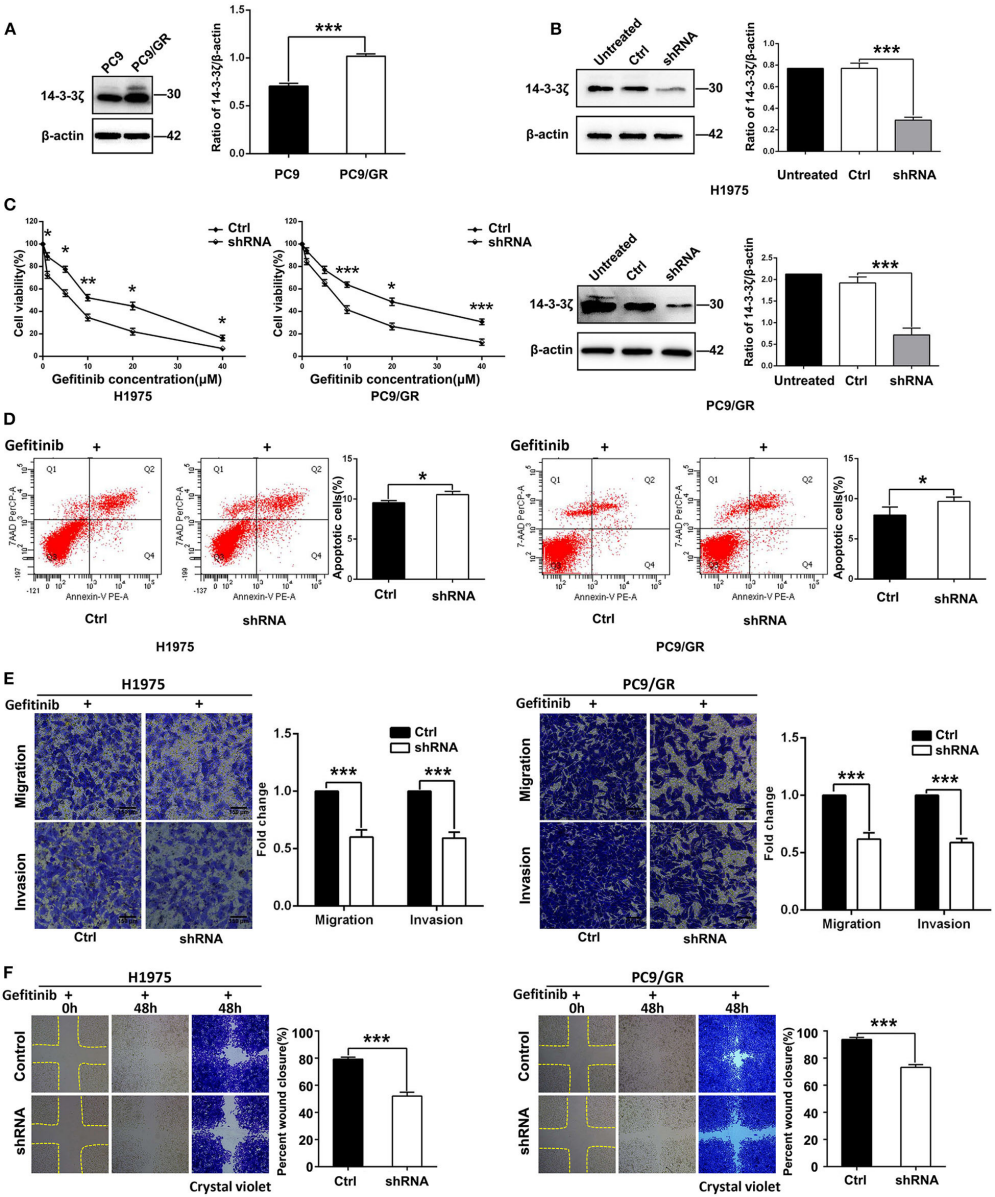
14-3-3ζ knockdown overcomes EGFR-TKI resistance in EGFR-mutant LUAD cells. **(A)** 14-3-3ζ protein expression levels in PC9 and PC9/GR cell lines, as determined by Western blot analysis. Histogram showing quantification of relative expression levels of 14-3-3ζ. β-actin was used as a loading control. **(B)** Western blot analyses of 14-3-3ζ protein expression levels in NCI-H1975 and PC9/GR cells and in cells transfected with scrambled-shRNA (Ctrl) or 14-3-3ζ-shRNA (shRNA). **(C)** H1975/14-3-3ζ-shRNA (left), PC9/GR/14-3-3ζ-shRNA (right), and corresponding vector control cells were treated with the indicated doses of gefitinib for 48 h, and cell viability was analyzed by a Cell Counting Kit-8 (CCK-8) assay. **(D)** Flow cytometric analysis of apoptosis in the indicated cells treated with gefitinib were assessed by Annexin V and 7-amino-actinomycin (7-AAD) staining. A representative flow profile is shown (left), and a summary of the percentage of Annexin V-positive cells is shown (right). **(E)** Transwell assays were conducted to assess EGFR-TKI-resistant cell migration and invasion after 14-3-3ζ knockdown in cells cultured in the presence of gefitinib compared with those of corresponding vector control cells (i.e., crystal violet staining of migratory and invasive cells). Original magnification, ×100; Scale bar 150 μm. **(F)** A wound healing assay was performed in the indicated cells as described in **(E)**. Original magnification, ×100. *p < 0.05, **p < 0.01, ***p < 0.001.

To identify whether 14-3-3ζ is relevant to EGFR-TKI resistance, Western blot analyses were performed to measure the 14-3-3ζ protein levels in the EGFR-TKI-sensitive LUAD cell line (PC-9) and its EGFR-TKI-resistant daughter cell line (PC-9/GR). A higher expression of the 14-3-3ζ protein was found in PC-9/GR cells than in PC-9 cells (Figure 3A). Next, in PC-9/GR and H1975 cells harboring an EGFR L858R/T790M mutation, expression vectors containing shRNA targeting 14-3-3ζ was used to decrease 14-3-3ζ expression. As shown in Figure 3B, compared to the control cells (Ctrl), 14-3-3ζ shRNA-transfected EGFR-TKI-resistant cells (14-3-3ζ-shRNA) displayed a decrease in the expression of 14-3-3ζ.

Knockdown of 14-3-3ζ sensitized PC-9/GR and H1975 cells when they were treated with different concentrations of gefitinib (Figure 3C). A similar finding was observed in flow cytometric analysis, wherein 14-3-3ζ-shRNA cancer cells exhibited a higher rate of apoptosis and were sensitive to gefitinib treatment (Figure 3D). To further investigate whether 14-3-3ζ-shRNA in combination with gefitinib has a better inhibitory effect on tumor cell motility, invasion, and migration than gefitinib alone, we performed Transwell and wound healing assays. Transwell assays revealed that knocking down 14-3-3ζ inhibited LUAD cell migration and invasion compared with control cells when combined with gefitinib (Figure 3E). Consistent with the Transwell assay results, the cells transfected with 14-3-3ζ-specific shRNA were slower to close the scratch wounds than the control cells when all cells were treated with gefitinib (Figure 3F). In summary, these *in vitro* data suggest that the combined 14-3-3ζ knockdown and gefitinib sensitizes resistant cells to EGFR-TKIs and overcomes resistance in EGFR-TKI-resistant cells.

### 14-3-3ζ Knockdown Inhibits EMT and BMP2/Smad/ID1 Signaling Activation

**Figure 4:**
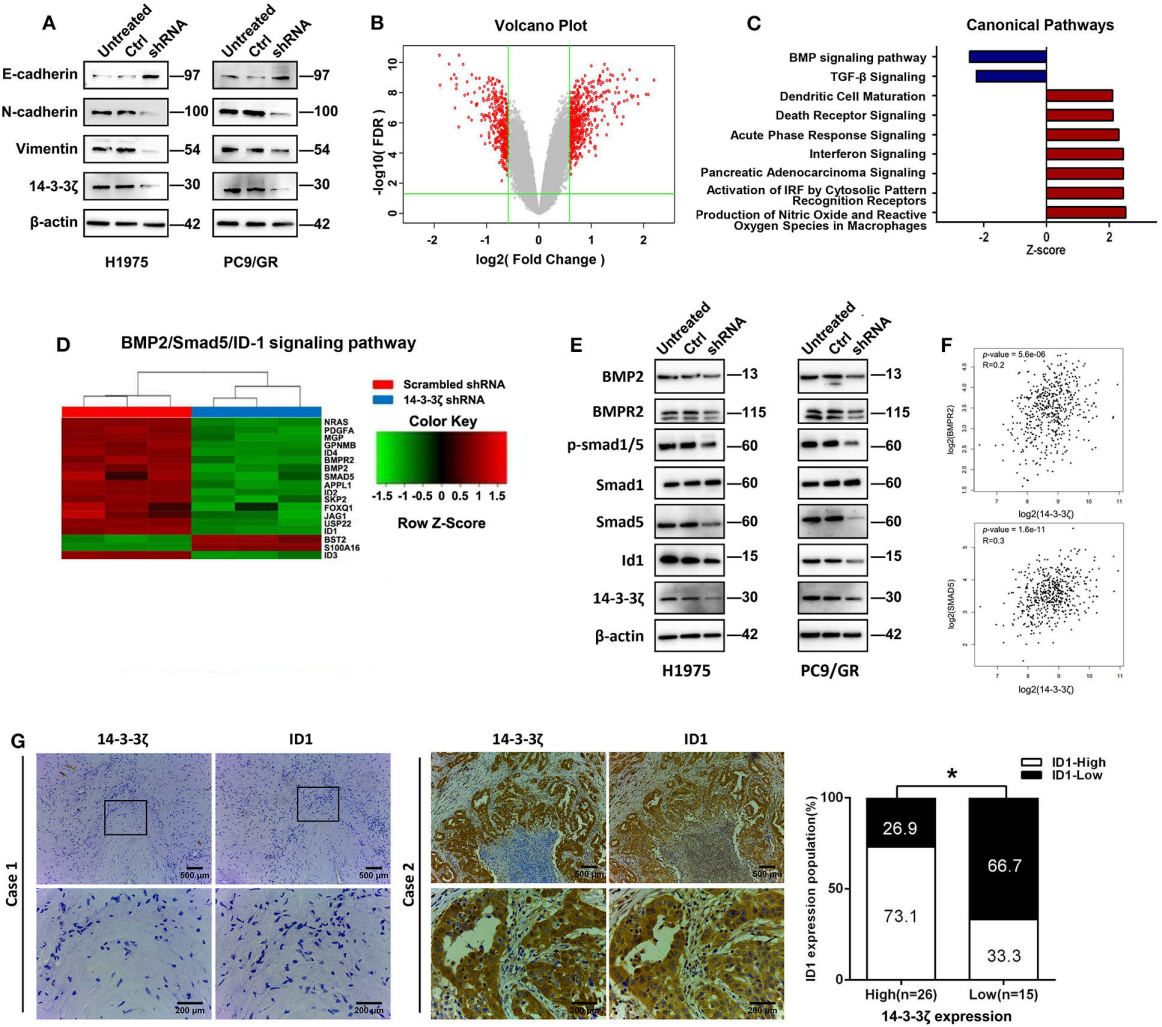
14-3-3ζ knockdown inhibits epithelial-to-mesenchymal transition (EMT) and the bone morphogenetic protein (BMP) pathway in EGFR-TKI-resistant cells. **(A)** Western blot analysis of E-cadherin, N-cadherin, and vimentin expression in EGFR-TKI-resistant cells and 14-3-3ζ-knockdown cells. β-actin was used as a loading control. **(B)** NCI-H1975 cells infected with lentivirus expressing either scrambled-shRNA or 14-3-3ζ-shRNA. Volcano plots are used to show genes with significant differences in data from two sets of samples. The abscissa is the multiple of the difference (logarithmic transformation with a base of 2), the ordinate is the significant false discovery rate (FDR; logarithmic transformation with a base of 10), and the red dots represent genes with significant differences. These genes were selected based on the absolute value of fold change ≥1.5 and FDR < 0.05. The gray dots represent other genes that have no significant difference. **(C)** The bar graph shows the significant enrichment of differentially expressed genes in the classical signaling pathways. According to IPA's internal algorithms and standards, a z-score ≥ 2 means that the pathway is significantly activated, and a z-score ≤ 2 means that the pathway is significantly inhibited. **(D)** Heatmap showing the differential expression of BMP2/Smad5/ID1 pathway gene signatures in NCI-H1975 cells infected with lentivirus expressing either scrambled-shRNA (blue) or 14-3-3ζ-shRNA (red). Genes and samples are listed in the rows and columns, respectively. A color key for the normalized expression data is shown at the top of the microarray heatmap (green represents downregulated genes; red represents upregulated genes). **(E)** NCI-H1975 and PC9/GR cells were transfected with 14-3-3ζ-shRNA (shRNA) or scrambled-shRNA (control) or were left untreated (untreated). The expression levels of BMP2, BMPR2, p-Smad1/5, Smad1, Smad5, and ID1 were determined using Western blotting. **(F)** The correlation between 14-3-3ζ and BMPR2 and Smad5 mRNA expression was identified by the TCGA database. **(G)** Representative images of immunohistochemical staining for 14-3-3ζ and ID1 in multiple sections of LUAD samples from patients. Patient 1 is representative of a patient with non-14-3-3ζ- overexpressing lung cancer, whereas Patient 2 is representative of a patient with 14-3-3ζ-overexpressing LUAD (left). There was a statistically significant correlation between the high expression of 14-3-3ζ and the high expression of ID1 in 41 LUAD tissues. The expression levels of 14-3-3ζ and ID1 were determined by immunostaining (right). Magnification, ×100; Scale bar = 500 μm. Magnification, ×400; Scale bar = 200 μm. *p < 0.05.

Next, we sought to explore the potential mechanism by which 14-3-3ζ knockdown regulates EGFR-TKI resistance. EMT is involved in phenotypic changes, which were identified as one of the EGFR-TKI resistance mechanisms (7, 9). The 14-3-3ζ depletion in EGFR-TKI-resistant cells led to the upregulation of epithelial markers (E-cadherin) and the downregulation of mesenchymal markers (N-cadherin and vimentin) (Figure 4A).

Furthermore, we determined global gene expression profiling in EGFR-TKI-resistant H1975 cells infected with a lentivirus expressing either scrambled shRNA or 14-3-3ζ-shRNA using microarray analysis. We identified 601 differentially expressed genes (*p* < 0.001 and absolute fold change > 1.5), which included 363 upregulated genes and 238 downregulated genes after 14-3-3ζ knockdown (Figure 4B). Using commercially available IPA software, we found that 14-3-3ζ knockdown affected a wide range of cellular functions (Supplementary Table 2) and canonical pathways (Supplementary Table 3). The absolute values of the z-score of canonical pathways >2 were ranked (Figure 4C). The BMP signaling pathway was notably downregulated by 14-3-3ζ-shRNA, suggesting that 14-3-3ζ could positively regulate the BMP signaling pathway. In detail, the BMP2/BMPR2/p-Smad(1/5)/ID1 axis was mediated by 14-3-3ζ knockdown, as shown by microarray technology (Figure 4D). The same finding was observed in EGFR-TKI-resistant cells using Western blotting (Figure 4E). In addition, a positive correlation among 14-3-3ζ, BMPR2, and Smad5 was observed in the LUAD samples from the TCGA database (Figure 4F).

To investigate the correlation between 14-3-3ζ expression and ID1 in LUAD patients with an EGFR mutation, the expression of ID1 was detected. The protein expression level of ID1 in these cases was also classified as low or high based on the intensity and proportion of positively stained cells in the IHC analysis (Supplementary Figure 3; Figure 4G, left). In line with our findings in the tumor cell lines, the distribution and intensity of 14-3-3ζ were positively correlated with ID1 in human EGFR-mutant LUAD specimens (Figure 4G, right).

### 14-3-3ζ Silencing Sensitizes EGFR-Mutant LUAD Cells to Gefitinib *in vivo*

**Figure 5:**
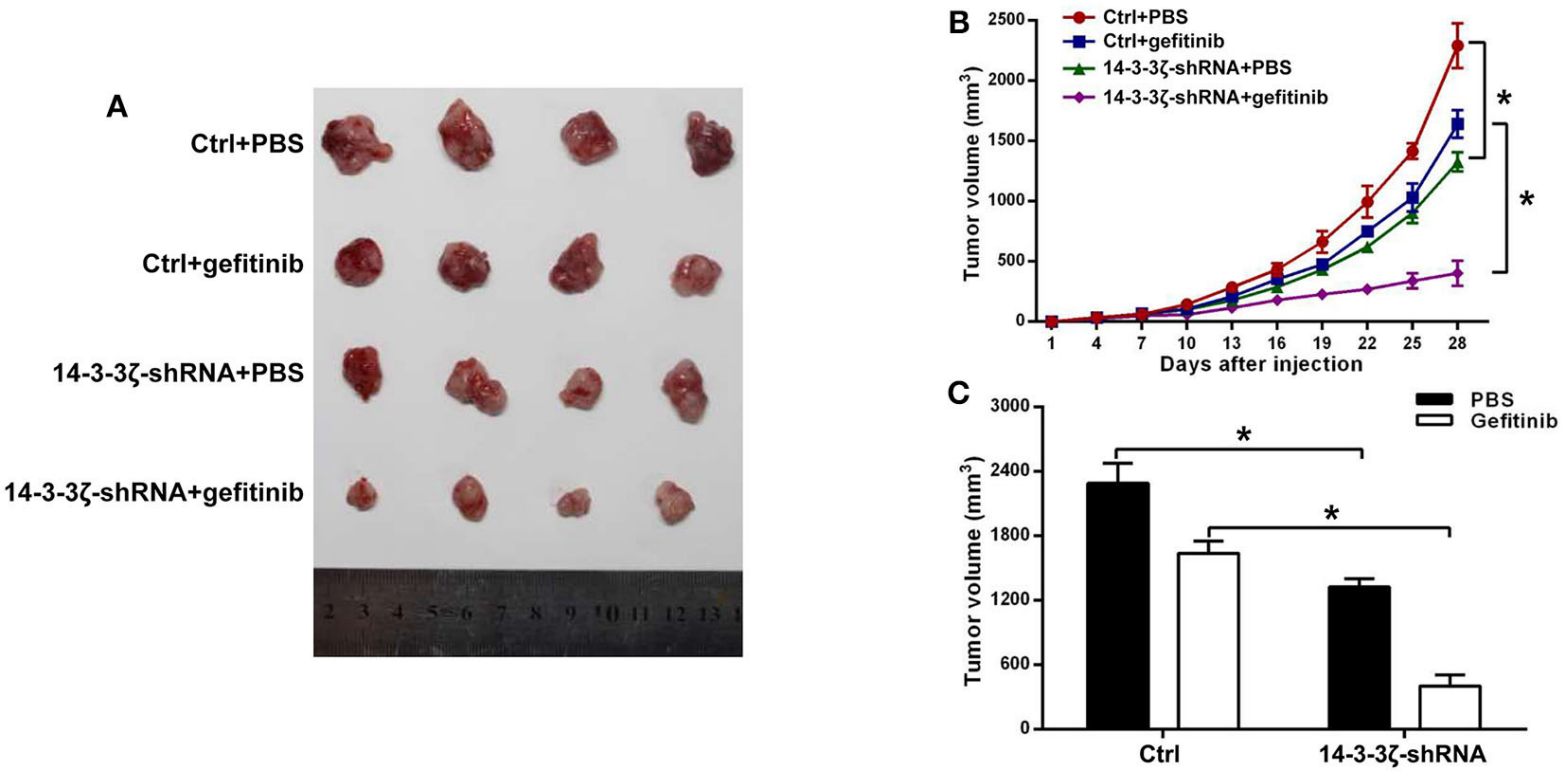
14-3-3ζ silencing enhances the sensitivity of EGFR-mutant LUAD to EGFR-TKIs in a mouse xenograft model. **(A)** Representative images of tumors at 28 days after inoculation using H1975/Ctrl or H1975/14-3-3ζ-shRNA cells treated with PBS or gefitinib. The Ctrl group consisted of mice inoculated with H1975/scrambled-shRNA cells. In the 14-3-3ζ-shRNA group, mice were inoculated with 14-3-3ζ-silenced H1975 cells. In the Ctrl + gefitinib group, mice were inoculated with control H1975 cells and treated with gefitinib. In the 14-3-3ζ-shRNA + gefitinib group, mice were inoculated with 14-3-3ζ-silenced H1975 cells and treated with gefitinib. **(B)** Tumor growth curves in nude mice injected with H1975/Ctrl or H1975/14-3-3ζ-shRNA cells treated with PBS or gefitinib. *N* = 4 for each group. **(C)** Tumor volumes at day 28 after inoculation. Left (black column), average tumor volumes at day 28 after inoculation with H1975/Ctrl or H1975/14-3-3ζ- shRNA cells in mice treated with PBS; right (white column), average tumor volumes at day 28 after inoculation of H1975/Ctrl or H1975/14-3-3ζ- shRNA cells in mice treated with gefitinib. *p < 0.05.

To determine the role of 14-3-3ζ in the sensitivity of EGFR-mutant LUAD cells to gefitinib *in vivo*, we injected 14-3-3ζ-shRNA or control shRNA into the ventral region of nonobese diabetic (NOD)-severe combined immunodeficient (SCID) mice when treated with gefitinib or PBS. Gefitinib treatment alone had little effect on the inhibition of tumor growth. Interestingly, 14-3-3ζ-shRNA alone was able to suppress the tumor volume (Figure 5, Supplementary Figure 4A) and tumor weight (Supplementary Figure 4B) and could further enhance these effects when combined with gefitinib. These results indicate that the knockdown of 14-3-3ζ sensitizes LUAD cells to gefitinib therapy *in vivo*.

## Discussion

EGFR-TKI therapies have shown diverse clinical benefits, and the overall responses range from 5 to 90% (41). Thus, the work to elucidate the cancer heterogeneity that impacts remission to EGFR-TKIs could be crucial to develop new therapeutic strategies with better efficacy for LUAD patients with EGFR-activating mutations (42).

In the present study, we first demonstrated that pancancer 14-3-3ζ expression was elevated and predicted unfavorable prognosis using the TCGA. We found that high 14-3-3ζ expression was significantly associated with advanced T stage, advanced TNM stage, the presence of lymph node metastasis, and poor treatment response to EGFR-TKI in LUAD patients with EGFR-activating mutations. Our study provides a molecular rationale for an unappreciated role of 14-3-3ζ in promoting EGFR-TKI resistance accompanied by EMT. Additionally, we showed that 14-3-3ζ positively regulated BMP2/Smad/ID1 signaling.

14-3-3ζ, as a central hub in signaling networks, exerts its functions by serine/threonine phosphorylation events involved in multiple cellular processes (11, 14, 43). The overexpression of 14-3-3ζ has been reported to contribute to the tumor progression of various malignancies by regulating critical processes, including the migration, cell cycle progression, apoptosis, differentiation, and metabolism of cancer cells (14–17, 19, 44). Consistently, we found that the expression of 14-3-3ζ, which is related to the product of oncogenes, was elevated in pancancer patients and correlated with worse clinical stages and survival of LUAD patients. Multiple lines of evidence reveal that 14-3-3ζ is a promising oncogenic predictor of progression and a prognostic biomarker in patients with lung cancer (45–49). Previous studies and our work emphasized the promising role of 14-3-3ζ as a novel target in lung cancer and pancancer therapies (11).

Here, we first demonstrated that suppression of 14-3-3ζ in LUAD cells enhanced the sensitivity to gefitinib. The molecular mechanisms that have been found to confer resistance to first- and second-generation EGFR-TKIs include the acquisition of the EGFR T790M mutation, MET amplification, HER-2 amplification, AXL activation, aberrant PI3K/AKT pathway, and phenotypic transformation, such as EMT (50–52). 14-3-3ζ was shown to regulate the PI3K/Akt pathway by enhancing Akt phosphorylation by binding to the p85α regulatory subunit of PI3K (53, 54). Whether 14-3-3ζ promotes EGFR-TKI resistance through the PI3K/Akt pathway needs to be further investigated in a future study.

Lung cancer cells with acquired resistance to gefitinib or osimertinib (AZD9291) show EMT characteristics, with a decrease in E-cadherin and an increase in mesenchymal markers and stemness without any EGFR secondary mutations (55). Considering the impact of EMT on EGFR-TKI resistance, we confirmed that 14-3-3ζ induced EMT in this study. In agreement with our results, increased expression of 14-3-3ζ promoted the EMT phenotype of cancer cells (16–18). 14-3-3ζ stabilized TGF-β RI, thereby activating the TGF-β/Smad pathway involved in EMT (18). In breast cancer, 14-3-3ζ cooperates with ErbB2 to promote the progression of ductal carcinoma *in situ* to invasive breast cancer by inducing EMT (17). In lung cancer, 14-3-3ζ prevented β-catenin ubiquitination and degradation, and subsequently induced EMT progression and invasiveness (16). Our results could help to improve therapeutic intervention, and 14-3-3ζ/EMT could serve as biomarkers in guiding the selection of patients who may particularly benefit from EGFR-TKIs.

To the best of our knowledge, we first illuminated the role of 14-3-3ζ in the regulation of BMP2/Smad/ID1 signaling. Based on previous studies, we reasoned that the possible molecular mechanism by which 14-3-3ζ regulates BMP2/Smad/ID1 signaling might be that 14-3-3ζ could alter the transcriptional activities of the BMP2 promoters. The zinc finger transcription factor Gli2 protein was reported to physically bind with the BMP2 promoter at specific regions that contain putative Gli-responsive elements, powerfully activating BMP2 gene expression (56). Interestingly, 14-3-3ζ was able to block Gli2 protein binding to its E3 ligase, thereby inhibiting Gli2 ubiquitination and increasing Gli2 stability (57, 58). As another BMP2 promoter, the NF-κB subunits p50 and p65 bound to the NF-κB response elements of the BMP2 gene, and NF-κB positively regulated BMP2 gene transcription (59). Previous studies have demonstrated that 14-3-3ζ could regulate the degradation of IκBα, allowing p65 to enter the nucleus (47). We hypothesized that 14-3-3ζ may activate NF-κB and subsequently promote BMP2 expression (47, 59, 60). Whether 14-3-3ζ-induced BMP2 expression depends on Gli2 or NF-κB needs further experimental dissection.

A limitation of this study is that only one shRNA was used to reduce 14-3-3ζ mRNA levels, with the goal of studying its function. Several studies have demonstrated that siRNA or shRNA are not always specific and can have many off-target effects (61, 62). High concentrations of shRNA can induce off-target effects and produce related phenotypes for certain shRNAs (61). Seed region sequence complementarity mediates widespread shRNA off-target transcript silencing (62). The use of more than one shRNA can prevent off-target effects (63), which will be considered and used in future work.

## Conclusion

In this study, we uncovered an unappreciated role of 14-3-3ζ in EGFR-TKI resistance *in vitro* and *in vivo*. In addition, 14-3-3ζ potentiated EMT and BMP2/Smad/ID1 signaling. Our findings suggest that targeting the 14-3-3ζ/BMP pathway/EMT could be a potential therapeutic strategy to reverse EGFR-TKI resistance in LUAD patients with acquired resistance.

## Data Availability Statement

The datasets presented in this study can be found in online repositories (64). The names of the repository/repositories and accession number(s) can be found below: NCBI Gene Expression Omnibus (GSE156435).

## Ethics Statement

The studies involving human participants were reviewed and approved by Ethics Committee of Harbin Medical University. The patients/participants provided their written informed consent to participate in this study. The animal study was reviewed and approved by Institutional Ethics Committee for the Administration of Laboratory Animals of Harbin Medical University.

## Consent for Publication

We obtained consent to publish this paper from all the participants of this study.

## Author Contributions

JC, YS, and XH performed the experiments, analyzed the data, and wrote the paper. HL and LC designed this research. JH and XC helped with the IHC assays. YC and XX helped with cell culture and Western blotting experiments. HL and YX critically revised the manuscript. All authors read and approved the final manuscript.

## Conflict of Interest

The authors declare that the research was conducted in the absence of any commercial or financial relationships that could be construed as a potential conflict of interest.

## Supplementary Material

Supplementary Figure 1 The experimental design and construction scheme of this study.

Supplementary Figure 2 Kaplan–Meier survival curves of OS and PPS comparing high and low 14-3-3ζ expression estimates of LUAD patients according to the Kaplan–Meier plotter database. Notes: The Affymetrix ID is 200641_s_at (YWHAZ).

Supplementary Figure 3 Representative IHC staining images of low expression and high expression of ID1 in LUAD tissues. Magnification, ×100; Scale bar = 500 μm.

Supplementary Figure 4 14-3-3ζ silencing enhances the sensitivity of EGFR-mutant LUAD to EGFR-TKIs in a mouse xenograft model. **(A)** Photographs of mice at 28 days after inoculation using H1975/Ctrl or H1975/14-3-3ζ-shRNA cells treated with PBS or gefitinib. **(B)** Tumor weight in the Ctrl + PBS, Ctrl + gefitinib, 14-3-3ζ-shRNA + PBS, and 14-3-3ζ-shRNA + gefitinib groups. ^*^*p* < 0.05.

Supplementary Table 1 Association between 14-3-3ζ expression and clinicopathological characteristics of LUAD patients with EGFR-activating mutations.

Supplementary Table 2 Statistically significant Diseases and Functions following 14-3-3ζ knockdown.

Supplementary Table 3 Statistically significant Canonical Pathways following 14-3-3ζ knockdown.

## Abbreviations

LUAD: lung adenocarcinoma
IHC: immunohistochemistry
EGFR-TKI: epidermal growth factor receptor-tyrosine kinase inhibitor
EMT: epithelial-to-mesenchymal transition
BMPs: bone morphogenetic proteins
ID1: inhibitors of differentiation 1
TGF-β: transforming growth factor-beta
CCK-8: Cell Counting Kit-8
NOD-SCID: nonobese diabetic severe combined immunodeficient.
