# Supplementary figures and images for: Targeting 14-3-3ζ Overcomes Resistance to Epidermal Growth Factor Receptor-Tyrosine Kinase Inhibitors in Lung Adenocarcinoma via BMP2/Smad/ID1 Signaling
Source: Front Oncol. 2020 Oct 5;10:542007. doi: 10.3389/fonc.2020.542007 (PMC7571474; doi:10.3389/fonc.2020.542007)

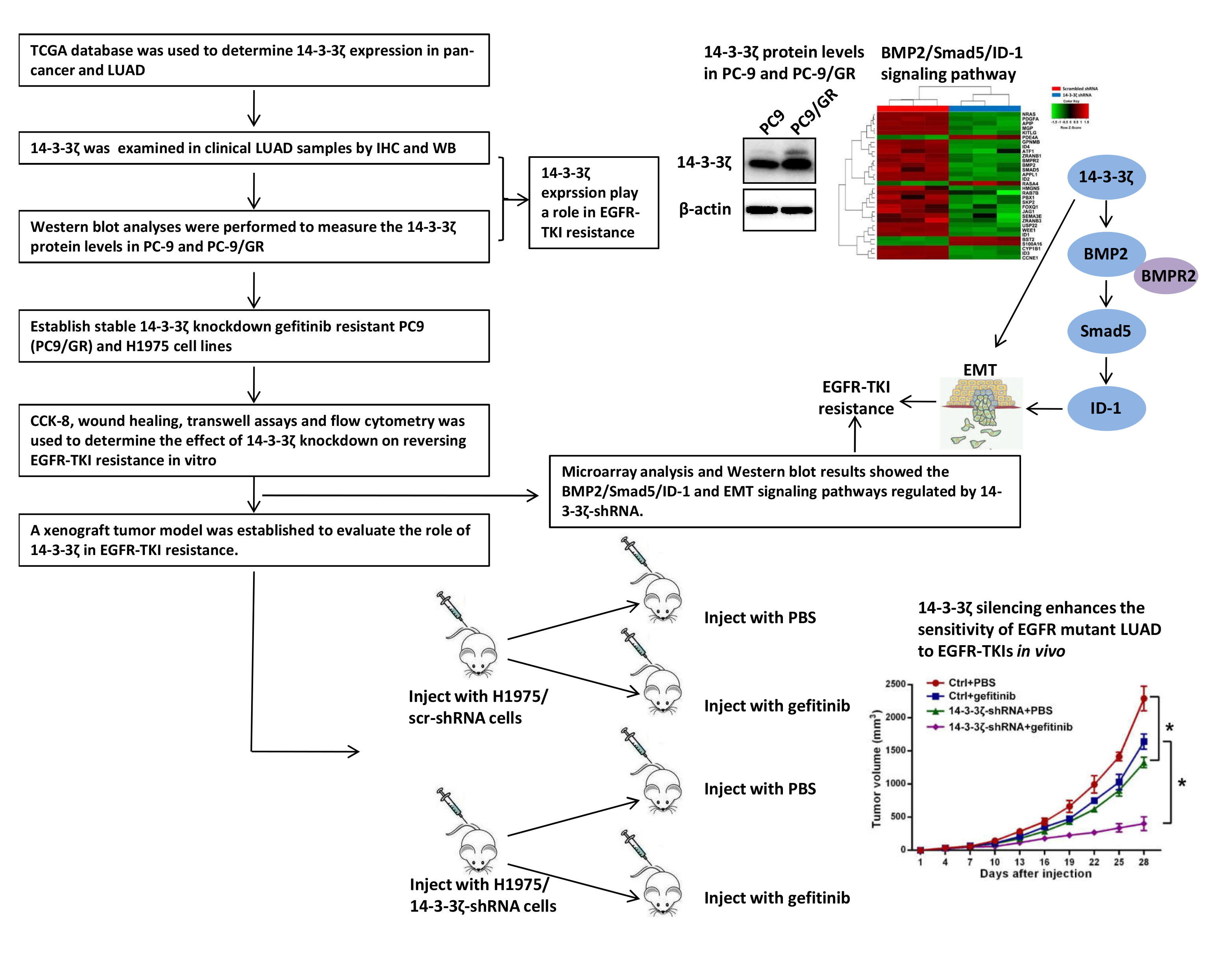

Supplement: Supplementary Figure 1 — The experimental design and construction scheme of this study. [file Image_1.JPEG]

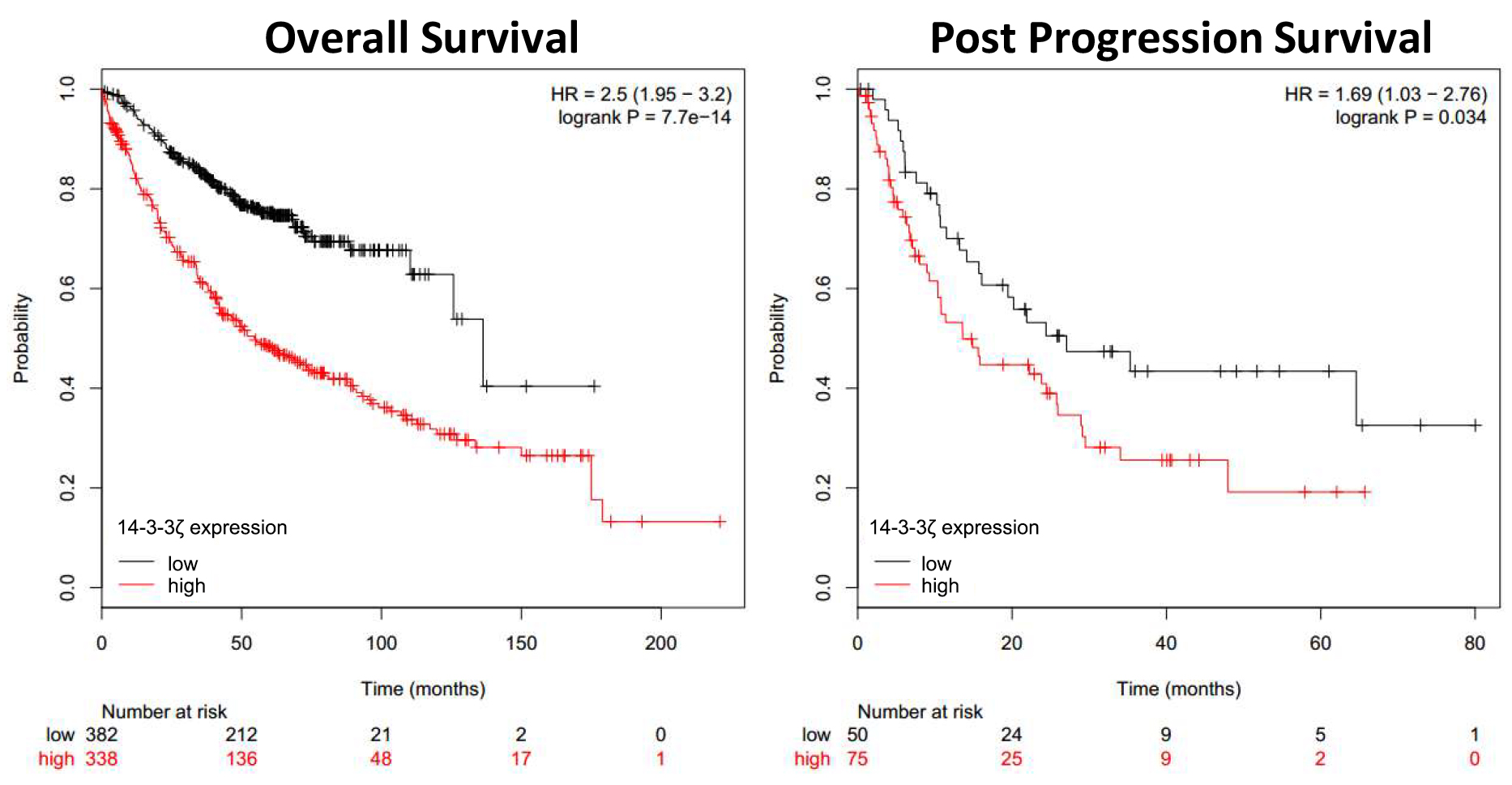

Supplement: Supplementary Figure 2 — Kaplan–Meier survival curves of OS and PPS comparing high and low 14-3-3ζ expression estimates of LUAD patients according to the Kaplan–Meier plotter database. Notes: The Affymetrix ID is 200641_s_at (YWHAZ). [file Image_2.JPEG]

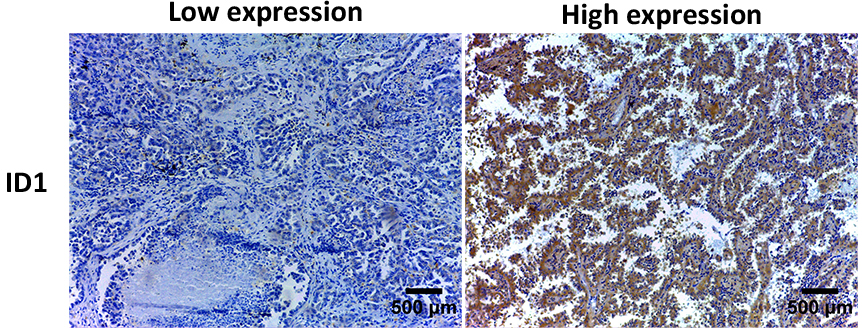

Supplement: Supplementary Figure 3 — Representative IHC staining images of low expression and high expression of ID1 in LUAD tissues. Magnification, ×100; Scale bar = 500 μm. [file Image_3.JPEG]

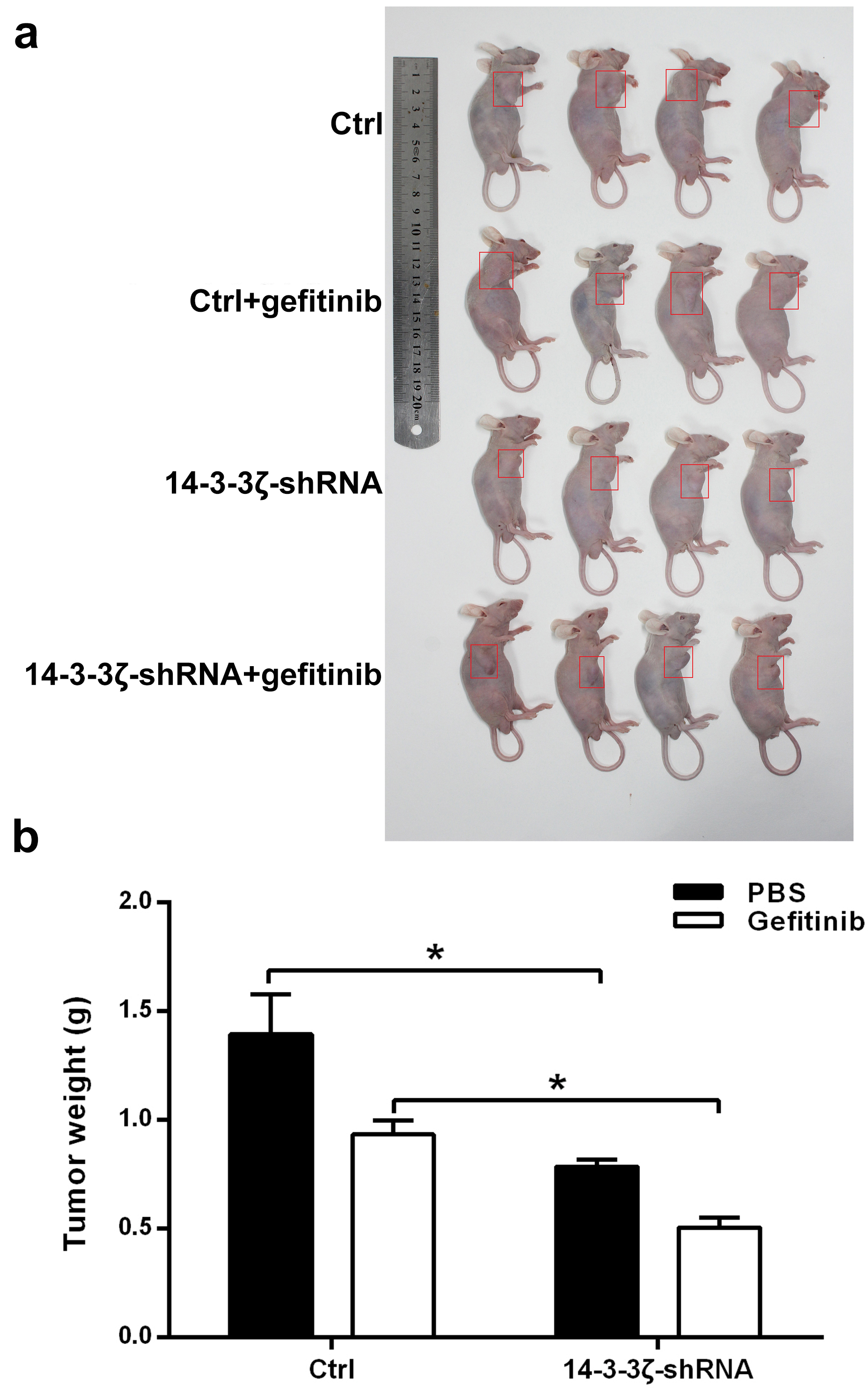

Supplement: Supplementary Figure 4 — 14-3-3ζ silencing enhances the sensitivity of EGFR-mutant LUAD to EGFR-TKIs in a mouse xenograft model. (A) Photographs of mice at 28 days after inoculation using H1975/Ctrl or H1975/14-3-3ζ-shRNA cells treated with PBS or gefitinib. (B) Tumor weight in the Ctrl + PBS, Ctrl + gefitinib, 14-3-3ζ-shRNA + PBS, and 14-3-3ζ-shRNA + gefitinib groups. *p < 0.05. [file Image_4.JPEG]
