# Supplementary material for: Targeting 14-3-3ζ Overcomes Resistance to Epidermal Growth Factor Receptor-Tyrosine Kinase Inhibitors in Lung Adenocarcinoma via BMP2/Smad/ID1 Signaling
Source: Front Oncol. 2020 Oct 5;10:542007. doi: 10.3389/fonc.2020.542007 (PMC7571474; doi:10.3389/fonc.2020.542007)
Supplement: Supplementary Table 1 — Association between 14-3-3ζ expression and clinicopathological characteristics of LUAD patients with EGFR-activating mutations. [file Table_1.docx]

| Table S1. Association between 14-3-3ζ expression and clinicopathological characteristics of LUAD patients with EGFR-activating mutations. | | | | |
| --- | --- | --- | --- | --- |
|  |  | 14-3-3ζ expression | |  |
| Variables | No. of Patients | Low | High | *p*-value |
| Gender |  |  |  | 0.923 |
| Male | 16 | 6 | 10 |  |
| Female | 25 | 9 | 16 |  |
| Age |  |  |  | 0.058 |
| ≥60 years | 25 | 12 | 13 |  |
| ＜60 years | 16 | 3 | 13 |  |
| Smoking status |  |  |  | 0.580 |
| Ever | 9 | 4 | 5 |  |
| Never | 32 | 11 | 21 |  |
| pT stage |  |  |  | 0.030*† |
| T1 | 20 | 11 | 9 |  |
| T2 | 10 | 3 | 7 |  |
| T3 | 11 | 1 | 10 |  |
| pTNM stage |  |  |  | 0.002*† |
| I | 18 | 12 | 6 |  |
| II | 15 | 2 | 13 |  |
| III | 8 | 1 | 7 |  |
| Lymph node metastasis |  |  |  |  |
| Present | 18 | 2 | 16 | 0.003* |
| Absent | 23 | 13 | 10 |  |
| EGFR-TKI response |  |  |  |  |
| sensitive | 21 | 11 | 10 | 0.031* |
| insensitive | 20 | 4 | 16 |  |
| ID1 |  |  |  | 0.013* |
| Low expression | 17 | 10 | 7 |  |
| High expression | 24 | 5 | 19 |  |

Abbreviations: LUAD=lung adenocarcinoma; EGFR-TKI=epidermal growth factor receptor-tyrosine kinase inhibitor; pT=pathological T stage; pTNM stage=tumor, node, metastasis (pathological stage). Ever: smoking at any time from the beginning of life. All clinicopathological characteristics except for EGFR-TKI response were classified when the patients underwent surgery. The EGFR-TKI insensitive group included patients with disease progression or stable disease without an extended (6 months) PFS, and the TKI-sensitive group included patients with a complete or partial response or stable disease with prolonged PFS (6 months). *p* value: the difference of clinicopathological characteristics between the 14-3-3ζ high expression group and low expression group. **p* < 0.05 was considered statistically significant. †The Fisher’s exact test.
